# Monogenic causes of familial short stature

**DOI:** 10.3389/fendo.2024.1506323

**Published:** 2024-12-19

**Authors:** Lukas Plachy, Petra Dusatkova, Shenali Anne Amaratunga, Vit Neuman, Zdenek Sumnik, Jan Lebl, Stepanka Pruhova

**Affiliations:** Department of Pediatrics, 2nd Faculty of Medicine, Charles University in Prague and Motol University Hospital, Prague, Czechia

**Keywords:** familial short stature, autosomal dominant short stature, genetics, growth plate, short stature

## Abstract

Genetic factors play a crucial role in determining human height. Short stature commonly affects multiple family members and therefore, familial short stature (FSS) represents a significant proportion of growth disorders. Traditionally, FSS was considered a benign polygenic condition representing a subcategory of idiopathic short stature (ISS). However, advancements in genetic research have revealed that FSS can also be monogenic, inherited in an autosomal dominant manner and can result from different mechanisms including primary growth plate disorders, growth hormone deficiency/insensitivity or by the disruption of fundamental intracellular pathways. These discoveries have highlighted a broader phenotypic spectrum for monogenic forms of short stature, which may exhibit mild manifestations indistinguishable from ISS. Given the overlapping features and the difficulty in differentiating polygenic from monogenic FSS without genetic testing, some researchers redefine FSS as a descriptive term that encompasses any familial occurrence of short stature, regardless of the underlying cause. This shift emphasizes the complexity of diagnosing and managing short stature within families, reflecting the diverse genetic landscape that influences human growth.

## Introduction

Genetic factors play a crucial role in determining human height with heritability exceeding 80% (1, 2). Consequently, growth failure is frequently present in multiple members of a family making familial short stature (FSS) one of the most common growth disorders (3). However, despite the widespread use of the term of FSS in routine clinical practice, it does not have a universally accepted definition.

Traditionally, FSS was considered a benign condition representing a subcategory of idiopathic short stature (ISS) – a child’s height less than 2 SD of the mean for a given age, sex and population but in concordance with midparental height (4). Polygenic inheritance is typically presumed. Moreover, socioeconomic situation may also contribute to FSS – short stature of a child and his/her parents corresponds with the situation of a population in a specific locality (5). Importantly, this definition presumes normal birth parameters and an absence of a secondary cause of short stature (e.g. systemic disease, endocrine disorder), chromosomal abnormality, monogenic condition or dysmorphic features including clinical signs of bone dysplasia (4).

Due to substantial progress in the genetic examination of short stature, it is now evident that monogenic short stature is more frequent than previously expected. Therefore, FSS may represent a monogenic condition inherited in an autosomal dominant (AD) manner as well (AD-FSS) (6, 7). Typically, one of the parents is substantially shorter than the other - the child’s height corresponds to the shorter parent’s height rather than the midparental height. In AD-FSS, body disproportionality, dysmorphic or other phenotypical signs corresponding to the specific genetic aetiology are assumed (8, 9). However, these clinical features might be subtle and frequently unrecognized (10). Due to these reasons and some others summarized in **Table 1**, AD-FSS might be very difficult to distinguish from classic FSS. Some authors therefore consider FSS only as a descriptive diagnosis of short stature occurring in multiple generations of a family regardless of the aetiology of the growth disorder or associated clinical features (11–14). This review covers the topic of FSS using this broader definition.

**Table 1:** Main differences between polygenic familial short stature and autosomal dominant short stature: the traditional approach and its pitfalls.

Parameters	Familial short stature (FSS)	AD familial short stature (AD-FSS)	Pitfalls
Aetiology	Polygenic	Monogenic	Genetic aetiology unknown in most cases
Parents‘ height	Both parents short, similar height SDS in both parents	Only one short parent or one parent substantially shorter than the second one	Tendency to look for a partner with similar height SDS.
Body disproportionality	Absent	May be present^+^	Children with AD primary growth plate disorders frequently have symmetric short stature or the asymmetry may appear in older age
Bone dysplasia signs	Absent	May be present^+^	Frequently absent in primary growth plate disorders
Birth parameters	AGA, SGA excludes FSS	SGA frequently present depending on the specific aetiology	Arbitrary boundary, multiple and heterogeneous aetiology of SGA
Growth hormone deficiency	Excludes FSS	May be rarely present depending on the aetiology	Diagnostics of growth hormone deficiency is highly unreliable

^+^In case of primary growth plate disorders. AD, autosomal dominant; AGA, appropriate for gestational age; FSS, familial short stature; SGA, small for gestational age.

## Main text

### Polygenic aetiology of familial short stature

Polygenic inheritance has long been presumed in FSS (9). Genome wide association studies have identified hundreds of genetic variants affecting human height. These variants are relatively common (allele frequency greater than 1% in the general population), each of them alone has only a small effect on human growth (around 1-4 mm) but may cause short stature if combined (15). Interestingly, several genetic variants have been described with relatively low allele frequency (<5%) and substantially higher individual effect (more than 10 times higher than the above-mentioned common variants). As these variants are frequently located in the genes whose pathogenic variants are known to cause monogenic growth disorders, they can be thought of as variants which create interplay between polygenic and monogenic inheritance (3).

### Monogenic aetiology of familial short stature (AD-FSS)

Short stature transmitted in the family in an AD manner can be caused by multiple mechanisms including primary growth plate disorders, GH deficiency/insensitivity or by the disruption of fundamental intracellular pathways (16). The sections that follow will offer insight into the specific mechanisms and phenotypic features potentially causing FSS.

#### AD-FSS caused by primary growth plate disorders

Primary growth plate disorders are frequent cause of AD-FSS (10, 17). In children with pathogenic variants in one of the genes essential for the correct function of the growth plate, disproportionate short stature (with relatively shorter limbs or with relatively shorter trunk) and other signs of bone dysplasia (e.g., bone deformities, scoliosis, brachydactyly) were traditionally believed to be apparent. However, in the past years, studies have proven that the phenotype of children with primary growth plate disorders is very variable including very mild phenotypical signs frequently detectable only via detailed anthropometric examination or short stature clinically unrecognizable from polygenic ISS (16, 18–20). The genetic aetiology of FSS caused by primary growth plate disorders are summarized in a **Supplementary Table**.

## SHOX-deficiency

The *SHOX* (Short Stature Homeobox Containing) gene located in the pseudoautosomal region of sex chromosomes encodes a nuclear transcriptional activator (SHOX protein) that promotes the differentiation of hypertrophic chondrocytes within the growth plate (21, 22). Deficiency of the SHOX protein (SHOX-D) is mostly (80%) caused by deletions involving the *SHOX* gene itself or its regulatory enhancer. Less frequently, SHOX-D is caused by point *SHOX* gene variants or by partial or complex *SHOX* duplication (21, 23). Heterozygous SHOX-D is a relatively frequent cause of a growth disorder with an estimated prevalence 2-15% of short children with a highly variable phenotype (21). Leri-Weil syndrome (LWS) is a condition with mesomelic shortening of the limbs (disproportionate short stature) and typical Madelung deformity of the forearm (bowing and shortening of the radius, subluxation of the distal ulna, pyramidal configuration of carpal bones). In milder forms, broadening of the forearm or its reduced ability to pronate and supinate may appear. Other clinical signs of LWS may include scoliosis, shortening of 4^th^ and 5^th^ metacarpals, high arched palate, micrognathia or muscular hypertrophy of the calves. Typical radiological finding in LWS is a triplet consisting of triangular shape of the distal radial epiphysis, carpal row pyramidalization and lucency of the ulnar side of the distal radius. However, auxological and radiological findings are frequently subtle or completely absent leading to a phenotype of idiopathic short stature, especially in younger children (21, 24–26). SHOX-D is one of the indications for GH therapy (27). Treatment efficacy seems to be equal compared to Turner syndrome leading to an average adult height gain of 7 cm (28).

## Natriuretic peptide receptor type B

Natriuretic peptide receptor type B encoded by the *NPR2* gene plays an important role in the paracrine regulation of the growth plate. The ligand (C-type natriuretic peptide) binding to the receptor, stimulates cell proliferation, cell differentiation and extracellular matrix synthesis (29, 30). Heterozygous pathogenic variants in the *NPR2* gene cause short stature (height -1.5 to -4.3 SD). In some children, clinical signs of bone dysplasia resembling LWS (e.g., disproportionate mesomelic short stature, high-arched palate, bone bowing, brachydactyly), but with no Madelung deformity present. However, most children with heterozygous pathogenic *NPR2* gene variants have short stature with no associated dysmorphic features, therefore they could remain undiagnosed. Interestingly, these mutations are responsible for 2-6% of idiopathic short stature including FSS (19, 31–33). Limited data suggest a good short-term response to GH therapy (19).

## Gain-of-function *FGFR3* gene variants

The *FGFR3* gene encodes fibroblast growth factor receptor type 3, a tyrosine kinase receptor that acts as a physiological negative regulator of skeletal growth by inhibiting the proliferation of chondrocytes. Gain-of-function variants in the *FGFR3* gene cause multiple disorders of varying severity, which are associated with short stature and variously expressed signs of bone dysplasia (34). Achondroplasia is the most common severe skeletal dysplasia with an incidence approximately 1:20000-1:30000 live births (35). It is characterized by severe short stature (average adult height 130 cm in men and 122 cm in women) (36) with disproportionately short arms and legs, macrocephaly, frontal bossing, mid-face hypoplasia, exaggerated lumbar lordosis, genua vara and bowing of the legs. Affected individuals frequently encounter the complication of foramen magnum stenosis including sleep-breathing disorders and are at an increased risk of infant death (37). The p.Gly380Arg variant is present in 98-99% individuals with achondroplasia. The disease is transmitted in an AD manner, however, approximately 80% of variants occur *de novo* (34, 37).

Hypochondroplasia is a milder bone dysplasia caused by gain-of-function variants in *FGFR3*. The prevalence of hypochondroplasia is unknown. Skeletal features are very similar to those seen in achondroplasia but tend to be milder. Body height usually varies between -3 to -2 SD (38). In some cases, clinical signs of bone dysplasia may be very subtle and the individuals with *FGFR3* gain-of-function variants have a phenotype corresponding to ISS (10, 39, 40).

## Growth plate collagenopathies

Collagens are the most abundant proteins in the human body playing an important structural role. They also participate in the regulation of cell growth, differentiation and migration by interacting with cellular receptors. Collagens II, IX, X and XI are present in the extracellular matrix of the growth plate (18, 41). The defects in individual collagen molecules cause various types of growth plate disorders frequently associated with short stature (41, 42). Variants in *COL2A1* gene are known to cause multiple syndromic bone dysplasias (e.g., Kniest dysplasia, Stickler syndrome) with substantial clinical heterogeneity. Bone dysplasia signs (e.g., disproportionate short stature, scoliosis, brachydactyly, metaphyseal abnormalities), distinct facial phenotype (e.g., cleft palate, mid-face hypoplasia), ocular complications (e.g., myopia, retinal detachment), sensorineural hearing loss, and joint deformities are common (18, 43, 44). Heterozygous variants in the *COL11A1* gene are known to cause Stickler syndrome, Marshal syndrome and phenotypes overlapping both disorders (45). Heterozygous pathogenic variants in collagen IX genes cause multiple epiphyseal dysplasia which can be associated with proximal muscle weakness (46, 47). Heterozygous variants in the *COL10A1* gene cause Schmid-type of epiphyseal chondrodysplasia characterized by short stature, widened growth plates and bowing of the long bones (48). Recently, heterozygous variants in growth plate collagen genes were shown to be a frequent cause of short stature with only subtle or absent syndromic features (18).

## Other extracellular matrix proteins defects

Aside from collagen, other proteins play an important role in the correct structure and function of the extracellular matrix of the growth plate. Among them, matrilin-3 (MATN3) and cartilage oligomeric matrix protein (COMP) are the most explored (49). Heterozygous variants in both *MATN3* and *COMP* genes (pathological intracellular accumulation of defective proteins explain the dominant negative effect (50, 51) leading to a phenotype of multiple epiphyseal dysplasia (MED), a relatively heterogeneous condition characterized by short stature, delayed and irregular epiphyseal ossification and early onset osteoarthritis (52, 53). Besides MED, heterozygous variants in COMP gene can cause pseudoachondroplasia with typical asymmetric short limbed short stature, limitation of joint function, bone deformities, scoliosis, spinal stenosis and brachydactyly (54).

## Defects in aggrecan

The *ACAN* gene encodes a proteoglycan aggrecan, one of the important components of the extracellular matrix of cartilaginous tissue (55). Pathogenic variants in the *ACAN* gene disrupt the correct structure and function of the growth plate and other cartilages (8). Most children with a heterozygous pathogenic variant in the *ACAN* gene have short stature associated with advanced bone age frequently leading to premature growth cessation. Some of the affected children may have some clinical signs of bone dysplasia including frontal bossing, midface hypoplasia, flat nasal bridge or brachydactyly. However, most children have short stature with no apparent body disproportionality or associated syndromic features (8, 55). As aggrecan is also present in the articular cartilage and intervertebral discs, early onset arthritis and intervertebral disc degenerative disease appears frequently in *ACAN* gene variants (8). Limited data show promising short-term response to GH therapy (8).

### AD-FSS caused by monogenic growth hormone deficiency

The clinical features of growth hormone deficiency are variable. Children with severe GHD may present in neonatal period with hypoglycemia or prolonged icterus. In older children, phenotypical signs typical for GHD (e.g., mid-face hypoplasia, truncal adiposity, thin sparce hair, high pitched voice, premature appearance) may be present. However, most children with GHD have no apparent clinical features besides short stature (56).

Autosomal dominant growth hormone deficiency (GHD) can be inherited by multiple different mechanisms. Impaired regulation of GH secretion might be caused by heterozygous variants in the *GHSR* gene encoding the ghrelin receptor (ghrelin is a hormone stimulating in GH release). Heterozygous variants in the *GHSR* gene can lead to the reduction of constitutive activity of the receptor, its intracellular retention or its binding affinity causing a variable degree of GHD (57, 58). Rarely, GHD might be caused by heterozygous mutations directly in the gene for GH. In this case, a splicing mutation in the *GH1* gene causes an overproduction of the 17.5 kD isoform of GH that is subsequently retained in the endoplasmic reticulum disrupting the secretion of GH (isolated growth hormone deficiency type II) and possibly other pituitary hormones as well (59). Moreover, GHD may be caused by heterozygous variants in genes affecting pituitary development (e.g., genes *SHH*, *PTCH1*, *GLI2*, *OTX2*, *SOX2*, *HESX1*, *PITX2*, *FGFR1*, *LHX4*, *PROKR2*) (60–63).

### AD-FSS caused by growth hormone insensitivity

Growth hormone mediates its effect by binding to the GH receptor (GHR). Heterozygous variants in *GHR* gene cause partial GH insensitivity (64). After GH is bound to GHR, the signal is transduced via a complex intracellular pathway stimulating the production of IGF-1 (insulin like growth factor type 1) (65). This intracellular signaling may be disrupted by mutations in *STAT5B* gene causing GH insensitivity associated with immune dysregulation that typically has an autosomal recessive inheritance. However, AD transmission has also been described (66). In blood, IGF-1 is stabilized by binding to the acid-labile subunit (ALS) and IGF binding protein type 3 (IGFBP-3) forming a ternary complex. Heterozygous variants in the *IGFALS* gene encoding the ALS can disrupt the ternary complex thus affecting the function of IGF-1 (65, 67). Heterozygous mutation in the *IGF1R* gene might lead to impaired IGF-1 sensitivity due to damage to its receptor (68).

### AD-FSS caused by Noonan syndrome and other RASopathies

The ubiquitous RAS/MAPK signaling pathway has a crucial role in controlling multiple functions of the human body including body height. Heterozygous variants of its components cause a heterogeneous group of disorders called RASopathies (69). In RASopathies, short stature is frequently associated with multiple syndromic features including typical facial appearance, chest deformity, cardiovascular abnormalities, psychomotor retardation or dysplasia of the lymphatic system (70). However, associated syndromic features might be mild and children with RASopathies might be concealed under the phenotype of ISS (71, 72). Besides Noonan syndrome and neurofibromatosis type 1, cardiofaciocutaneous syndrome, Costello syndrome or Legius syndrome are other examples of RASopathies. The genes whose variants are responsible for RASopathies include *PTPN11*, *SOS1*, *RAF1*, *RIT1* or *KRAS* (73). Studies have proven a positive effect of GH treatment in children with Noonan syndrome (average increment of height increment 1.4 SD) (74, 75). Noonan syndrome is included among the diagnostic indications for GH treatment in both Europe and the USA (70, 76).

### How to identify a child with AD-FSS

During the first examination of a child with FSS a possibility of AD-FSS should be considered. Apart from the detailed examination of a child, we recommend evaluating both parents’ height ideally with their body proportionality, possible dysmorphic features, orthopedic or ophthalmological problems, fracture history, presence of heart diseases or other signs that might be associated with a monogenic disorder. In some cases, a detailed pedigree with the height and phenotype of more distant relatives might also be useful. In case a child corresponds to the diagnosis of AD-FSS (see **Table 1**), it is advisable to indicate detailed genetic examination to determine if there is a monogenic cause for FSS. Even children whose phenotype indicates rather a polygenic aetiology of FSS might have AD-FSS. In this case, genetic examination might also be considered, especially in children with more severe growth disorder.

In the near future, facial recognition systems using artificial intelligence might be a highly effective tool for detecting even minor features typical for a specific genetic disorder associated with short stature. Multiple diseases including endocrine and metabolic disorders and rare genetic syndromes have several specific facial characteristics. Traditionally, evaluating specific facial features were a part of diagnosing genetic disorders, however, the efficacy highly depends on the experience of the physician or clinical anthropologist. Automatic recognition systems have substantial potential to save time, overcome the limited availability of experts skilled in detecting subtle phenotypic features typical for specific disorders or even detect very subtle changes hardly recognizable to the human eye (77–80).

## Conclusions

Familial short stature is a heterogeneous entity with a substantial proportion of monogenic causes, which may have subtle phenotypic features, possible co morbidities and variable response to GH therapy. Genetic examination is frequently the only way to differentiate AD-FSS from polygenic FSS and should be considered in children with FSS. Elucidating the genetic diagnosis not only explains the cause of growth disorder in the family and enables a focus on possible hidden co morbidities associated with genetic findings, but is important from a scientific perspective as well. Studies focusing on the genetic examination of FSS have immense potential to further improve our knowledge regarding the aetiology of short stature in general. Moreover, due to the anticipated high detection rate of various genetic conditions, these results might provide crucial data for research aiming to elucidate genetic disorder-specific reaction to GH treatment.
