# Supplementary material for: Monogenic causes of familial short stature
Source: Front Endocrinol (Lausanne). 2024 Dec 19;15:1506323. doi: 10.3389/fendo.2024.1506323 (PMC11693446; doi:10.3389/fendo.2024.1506323)
Supplement: Supplementary file 1 [file Table1.docx]

| **Gene** | **Clinical features** | **OMIM reference number** | **Manuscript pages** |
| --- | --- | --- | --- |
| *SHOX* | 1) **Léri-Weil syndrome:** disproportionate short stature, Madelung forearm deformity, scoliosis, short 4^th^ and 5^th^ metacarpals, high arched palate, micrognathia, muscular hypertrophy of the calves  RTG: triangular distal radial epiphysis, carpal row pyramidalization, lucency of distal ulnar radius  2) **Idiopathic short stature** | 127300  NA |  |
| *NPR2* | 1) **Short stature with signs of bone dysplasia:** disproportionate mesomelic short stature, brachydactyly, leg bowing, high arched palate  2) **Idiopathic short stature** | 616255  NA |  |
| *FGFR3* | 1) **Achondroplasia**: severe disproportionate short stature (average adult height 130 cm in men, 122 cm in women), very short arms and legs, macrocephaly, frontal bossing, mid-face hypoplasia, exaggerated lumbar lordosis, genua vara, leg bowing, foramen magnum stenosis, repeated otitis media acuta, conductive hearing loss  2) **Hypochondroplasia**: less severe short stature (-3 to -2 SD), milder phenotype resembling achondroplasia  3) **Idiopathic short stature** | 100800  146000  NA |  |
| *COL2A1* | 1) **Achondrogenesis type 2**: micromelic dwarfism, incomplete ossification of vertebral bodies, small chest, hypoplastic pelvis, epiphyseal dysplasia, cleft palate, lethal before or shortly after birth  2) **Platyspondylic skeletal dysplasia, Torrance type:** severe micromelic dwarfism, platyspondyly, hypoplastic thorax, short ribs with anterior cupping, lumbar hyperlordosis, hypoplastic pelvis, brachydactyly, metaphyseal changes, cleft palate, micrognathia, prominent abdomen, frequently lethal before or shortly after birth  3) **Spondyloperiphereal dysplasia**: micromelic dwarfism, platyspondyly, hip dysplasia, brachydactyly, midface hypoplasia, ocular complications, hearing loss  4) **Spondyloepiphyseal dysplasia congenita**: short-trunk dwarfism, odontoid hypoplasia, cervical spine subluxation, scoliosis, hyperkyphosis, excessive lumbar lordosis, coxa vara, genua valga, clubfoot, pes planus, metaphyseal changes, midface hypoplasia, cleft palate, micrognathia, sensorineural hearing loss, ocular complications, hypoplastic abdominal muscles, inguinal hernia  5) **Spondyloepimetaphyseal dysplasia, Strudwick type**: short-trunk/short-limbed dwarfism, odontoid hypoplasia, scoliosis, hyperlordosis, pectus carinatum, hip joint stiffness, coxa vara, genua valga, clubfoot, midface hypoplasia, cleft palate, ocular complications  6) **Kniest dysplasia**: Short-trunk dwarfism, joint contractures, other arthropathies, kyphoscoliosis, long bone deformities, platyspondyly with coronal clefts, midface hypoplasia, cleft palate, conductive hearing loss, myopia  7) **Multiple epiphyseal dysplasia with myopia and deafness**: Dwarfism, epiphyseal dysplasia with minor abnormalities of the phalanges, femoral heads and spine, Mid-face hypoplasia, depressed nasal bridge and small nose, cleft palate, sensorineural deafness, ocular complications  8) **Stickler syndrome**: normal stature, osteoarthritis, joint hypermobility, midface hypoplasia, cleft palate, sensorineural hearing loss, ocular complications  9) **Czech dysplasia**: normal stature, progressive pseudorheumatoid arthritis, platyspondyly, short third/fourth toe, hearing loss  10) **Osteoarthritis with mild chondrodysplasia**: normal stature, vertebral dysplasia, joint pain, joint stiffness, subchondral stenosis, osteophytes  11) **Idiopathic short stature** | 200610  151210  271700  183900  184250  156550  132450  108300  609162  604864  NA |  |
| *COL11A1* | 1) **Stickler syndrome**: normal stature, osteoarthritis, joint hypermobility, midface hypoplasia, cleft palate, sensorineural hearing loss, ocular complications  2) **Marshal syndrome**: short stature, thickening of calva, mild platyspondyly, midface hypoplasia, facial dysmorphism, ocular complications, hearing loss, ectodermal dysplasia, missing teeth  3) **Idiopathic short stature** | 604841  154780  NA |  |
| *COL11A2* | 1) **Non-ocular Stickler syndrome**: normal stature, osteoarthritis, midface hypoplasia, cleft palate, facial dysmorphism, sensorineural hearing loss, no ocular complications  2) **Fibrochondrogenesis**: rhizomelic dwarfism, small bell-shaped thorax, platyspondyly, midface hypoplasia, cleft palate, facial dysmorphism, omphalocele, lethal in prenatal or early postnatal period  3) **Idiopathic short stature** | 604841  614524  NA |  |
| *COL9A1, COL9A2, COL9A3* | 1) **Multiple epiphyseal dysplasia**: short stature, epiphyseal dysplasia, early-onset osteoarthritis, irregular end plates of vertebral bodies, Schmorl’s nodes, narrowed disk spaces, proximal muscle weakness (in *COL9A3*) | 614135 |  |
| *COL10A1* | 1) **Metaphyseal dysplasia**, Schmid type: short stature, joint deformities, lower limb deformities, metaphyseal changes | 156500 |  |
| *COMP* | 1) **Multiple epiphyseal dysplasia**: short stature, delayed and irregular ossification of the epiphyses, coxa vara, genua valga, brachydactyly early-onset osteoarthritis  2) **Pseudoachondroplasia**: disproportionate short stature with short limbs, limitations of joint function, joint laxity, scoliosis, spinal stenosis, bone deformities, brachydactyly, delayed ossification, platyspondyly | 132400  177170 |  |
| *ACAN* | 1) **Spondyloepiphyseal dysplasia, Kimberley type**: proportionate short stature, stocky appearance, severe progressive osteoarthritis, prominent end-plate irregularity, vertebral bodies sclerosis  Adult height median -2.9 SD (range -5.9 to -0.9 SD). Birth parameters usually in the lower part of the normal range. Frequently advanced bone age. Mostly normal head circumference.  2) **Idiopathic short stature** | 608361  NA |  |
| *MATN3* | 1) **Multiple epiphyseal dysplasia**: short stature, delayed and irregular ossification of the epiphyses, coxa vara, genua valga, brachydactyly early-onset osteoarthritis | 607078 |  |

**References**

1. Deng H, Huang X, Yuan L. Molecular genetics of the COL2A1-related disorders. Vol. 768, Mutation Research - Reviews in Mutation Research. 2016. p. 1–13.

2. Majava M, Hoornaert KP, Bartholdi D, Bouma MC, Bouman K, Carrera M, et al. A report on 10 new patients with heterozygous mutations in the COL11A1 gene and a review of genotype-phenotype correlations in type XI collagenopathies. American Journal of Medical Genetics, Part A. 2007.

3. Tompson SW, Bacino CA, Safina NP, Bober MB, Proud VK, Funari T, et al. Fibrochondrogenesis results from mutations in the COL11A1 type XI collagen gene. Am J Hum Genet. 2010;

4. Vuoristo MM, Pappas JG, Jansen V, Ala-Kokko L. A stop codon mutation in COL11A2 induces exon skipping and leads to non-ocular stickler syndrome. Am J Med Genet. 2004;

5. Sirko-Osadsa DA, Murray MA, Scott JA, Lavery MA, Warman ML, Robin NH. Stickler syndrome without eye involvement is caused by mutations in COL11A2, the gene encoding the α2(XI) chain of type XI collagen. J Pediatr. 1998;

6. Melkoniemi M, Brunner HG, Manouvrier S, Hennekam R, Superti-Furga A, Kaariainen H, et al. Autosomal Recessive Disorder Otospondylomegaepiphyseal Dysplasia Is Associated With Loss-of-Function Mutations in the COL11A2 Gene. Am J Hum Genet. 2000;

7. Tompson SW, Faqeih EA, Ala-Kokko L, Hecht JT, Miki R, Funari T, et al. Dominant and recessive forms of fibrochondrogenesis resulting from mutations at a second locus, COL11A2. Am J Med Genet Part A. 2012;

8. Van Camp G, Snoeckx RL, Hilgert N, van den Ende J, Fukuoka H, Wagatsuma M, et al. A new autosomal recessive form of Stickler syndrome is caused by a mutation in the COL9A1 gene. Am J Hum Genet. 2006;

9. Czarny-Ratajczak M, Lohiniva J, Rogala P, Kozlowski K, Perälä M, Carter L, et al. A Mutation in COL9A1 Causes Multiple Epiphyseal Dysplasia: Further Evidence for Locus Heterogeneity. Am J Hum Genet. 2001;

10. Bonnemann CG, Cox GF, Shapiro F, Wu J-J, Feener CA, Thompson TG, et al. A mutation in the alpha 3 chain of type IX collagen causes autosomal dominant multiple epiphyseal dysplasia with mild myopathy. Proc Natl Acad Sci. 2000;

11. Mäkitie O, Susic M, Ward L, Barclay C, Glorieux FH, Cole WG. Schmid type of metaphyseal chondrodysplasia and COL10A1 mutations - Findings in 10 patients. Am J Med Genet. 2005;
